# Differing sizes of bullet entrance holes in skin of the anterior and posterior trunk

**DOI:** 10.1007/s00414-022-02879-x

**Published:** 2022-08-25

**Authors:** D. Geisenberger, M. Große Perdekamp, S. Pollak, A. Thierauf-Emberger, V. Thoma

**Affiliations:** Institute of Forensic Medicine, Faculty of Medicine, University of Freiburg, Albertstraße 9, 79104 Freiburg, Germany

**Keywords:** Bullet entrance hole, Skin defect, Size of gunshot entrance, Dorsal skin, Abdominal skin

## Abstract

The aim of the present study was to establish whether the size (diameter and area) of bullet entrance holes in skin varies between distant shots to the anterior and posterior trunk, respectively, when using the same ammunition (in concreto pistol cartridges 9 mm Luger). For that purpose, specimens of porcine skin from the belly region and the back were taken (10 samples each) and shot at from a distance of 1.6 m. The entrance holes were photo-documented under standardised conditions. After image processing for contrast enhancement, the maximum diameter and the area of each skin defect were measured automatically by means of an image analysis system. Both size parameters differed significantly depending on the body region affected. On the back with its comparatively thick dermis, the skin defects were considerably smaller than those on the ventral trunk where the corium is less thick. This difference can be explained by the fact that the elastic properties of skin are strongly determined by the connective tissue which is especially rich in fibres. The study results were consistent with the authors’ casework experience and support the assumption that the entrance site of gunshot wounds has a major influence on the size of the bullet hole in skin.

## Introduction

The medical and forensic assessment of gunshot injuries is known to be prone to errors [1, 2]. In shots from rifled weapons, this applies to the differentiation of entrance and exit wounds, the number of hits, the firing distance as well as the type and calibre of ammunition. A crucial requirement for the correct evaluation of firearm deaths is the identification of bullet entrance wounds. In distant shots, the morphological diagnosis is based on the following features [3, 4]: (1) a mostly roundish tissue defect at the perforation site (bullet hole); (2) a ring of dirt (bullet wipe) in unclothed body regions being the primary target; (3) an abrasion collar surrounding the central skin defect. In forensic pathology, distant shots are defined by the absence of visible gunshot residues in the depth of the entrance wound and on the skin surrounding it.

More than a hundred years ago, it was already emphasised in textbooks that the mere dimensions of an entrance wound do not allow a reliable statement about the bullet’s calibre [5]. In the first edition of his classical monograph, Di Maio [3] wrote: “The caliber of the bullet that caused an entrance wound cannot be determined by the diameter of the entrance.”

The factors influencing the size of the permanent entrance hole are manifold: apart from some bullet parameters (calibre, design, velocity, any deformation or tumbling), the physical properties of the perforated skin are decisive, especially its elasticity in terms of a reversible deformation under stress. In spite of the many variables, the forensic pathologist is often expected to comment on the calibre in cases of perforating (“through-and-through”) gunshot injuries where the projectile is not retained in the victim’s body and is therefore not available for direct examination.

**Fig. 1:**
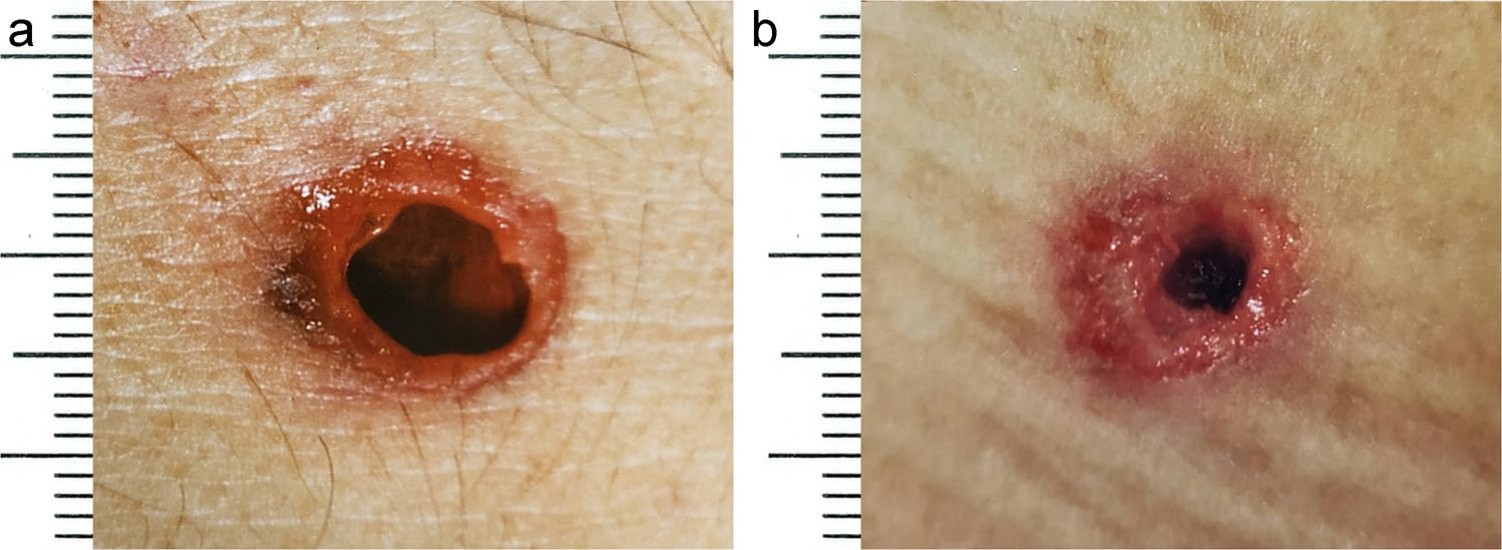
Gunshot entrance wounds from distant shots using pistol cartridges with round-nosed bullets cal. 9 mm Luger. The skin hole is comparatively large on the frontal trunk (**a**) and considerably smaller on the back (**b**)

Casework experience teaches us that distant (far-ranged) shots fired from the same gun and using the same ammunition can cause bullet entrance holes of varying size in different body regions (Fig. 1). The discrepancies seem to be particularly distinct, when wounds on the anterior and posterior trunk are compared. In such cases, different diameters of the skin defects may wrongly lead to the assumption that the affected person was hit by bullets of various calibres. In order to check the thesis that there is a correlation between the skin properties in a definite body region and the size of a bullet hole located there, a study using porcine skin as target was performed.

## Material and methods

**Fig. 2:**
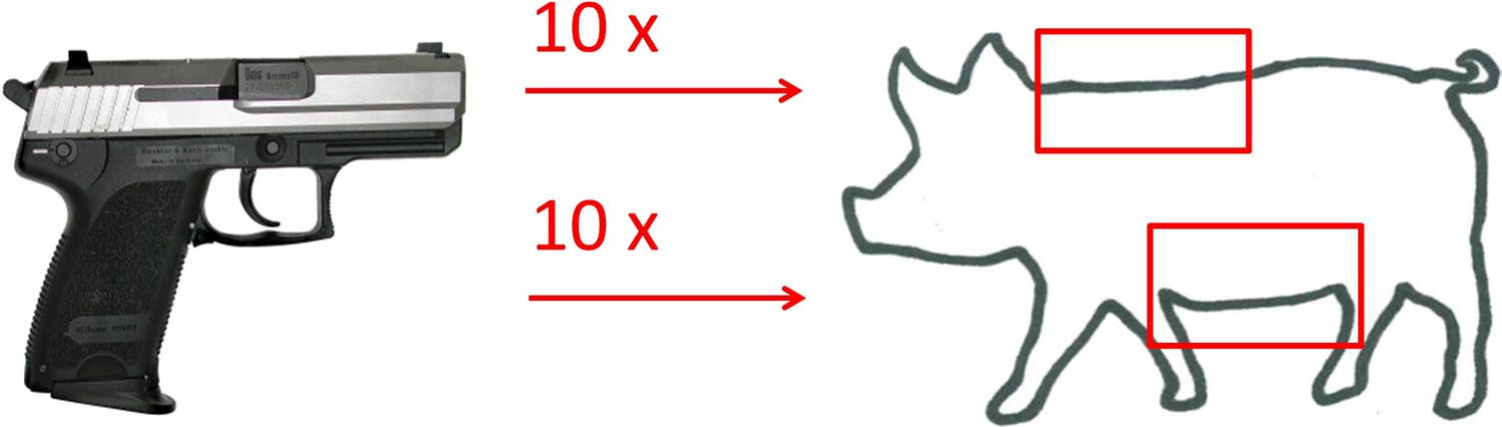
The experimental design comprised 10 distant shots to porcine skin from both the back and belly region

Fresh skin samples from a slaughtered German Landrace pig (age 6 months, weight 110 kg) were taken together with the underlying subcutis. Slaughter took place for regular meat production and thus was independent of the present research project. The hog hair had already been removed by the butcher. The specimens from both the back and belly region measured 20 × 30 cm (Fig. 2). First, the epidermis was dyed blue by incubating each piece with haemalum [6]. Subsequently, the superficially dyed integument was fixed in a frame so that the edge lengths corresponded with the original dimensions of the resected skin pieces.

The test shots were fired from a semi-automatic pistol Heckler & Koch P9 cal. 9 × 19 mm Parabellum (also known as 9 mm Luger). The cartridges (Geco™, RUAG Ammotec, Bern, Switzerland) were fitted with full-metal jacket round nose bullets. The shooting distance was 1.6 m and the direction of the shot was orthogonal to the target. Altogether, 10 shots each were fired to the specimens from the back and the belly region.

All bullet entrance sites were photographed separately together with a metric scale using a digital camera (Nikon D300, Tokyo, Japan) mounted on a tripod. Analogous to the procedure described in a previous study [6], the digital photos were processed by means of an image editing program (Ulead PhotoImpact, Softonic International, Barcelona, Spain) with the objective of optimising contours and contrast. As a result, the bullet holes appeared red whereas the surrounding skin was dark blue. Based on the contrast, the metric dimensions of the entrance holes were determined automatically employing an image analysing system (GSA GmbH, Rostock, Germany). In this way, both the maximum diameters, the area sizes of the bullet holes as well as the mean values and the standard deviations were calculated.

For statistical analysis, Student’s *t*-test for independent samples was used to compare the two study groups (skin of the belly region vs. the back). Regarding the area sizes, Welch’s *t*-test was used to check whether the samples have unequal variances.

In order to assess the dermal thickness in the respective body regions, representative tissue samples were fixed, dehydrated and embedded in paraffin wax. After being cut into thin slices, the specimens underwent the usual procedure applied in H&E staining.

## Results

**Fig. 3:**
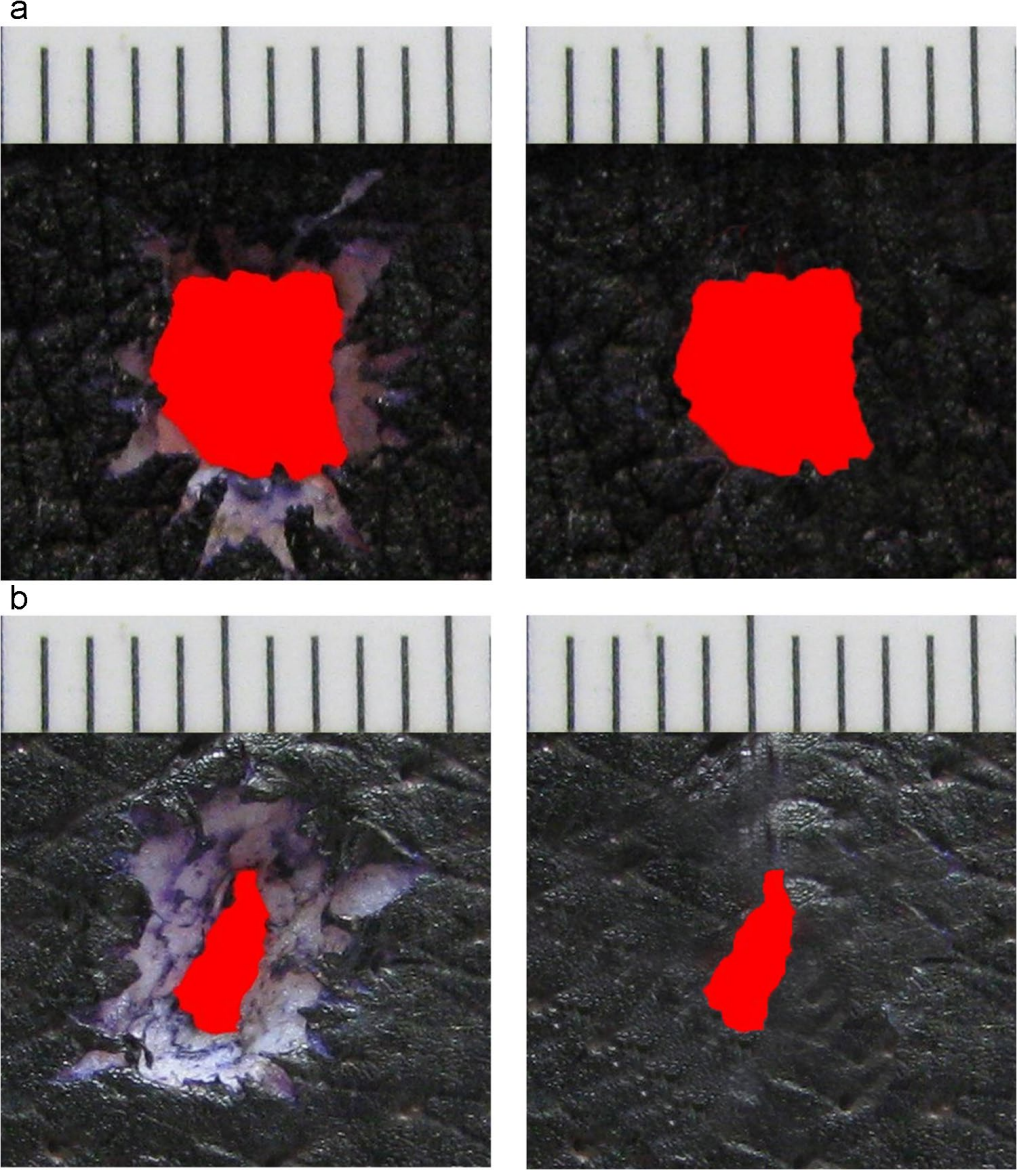
Typical examples for experimental gunshot entrance wounds in porcine skin. The dark blue colour of the epidermis is due to prior dyeing with haemalum. There is a distinct difference in size between the gunshot holes in the abdominal (**a**) and dorsal (**b**) skin. The central defects are surrounded by epidermis-free abrasion-rings appearing pale-grey. After being processed by an image editing program, the actual skin holes assumed a homogenous red colour, so that the areas could be determined by applying an analysing system

**Fig. 4:**
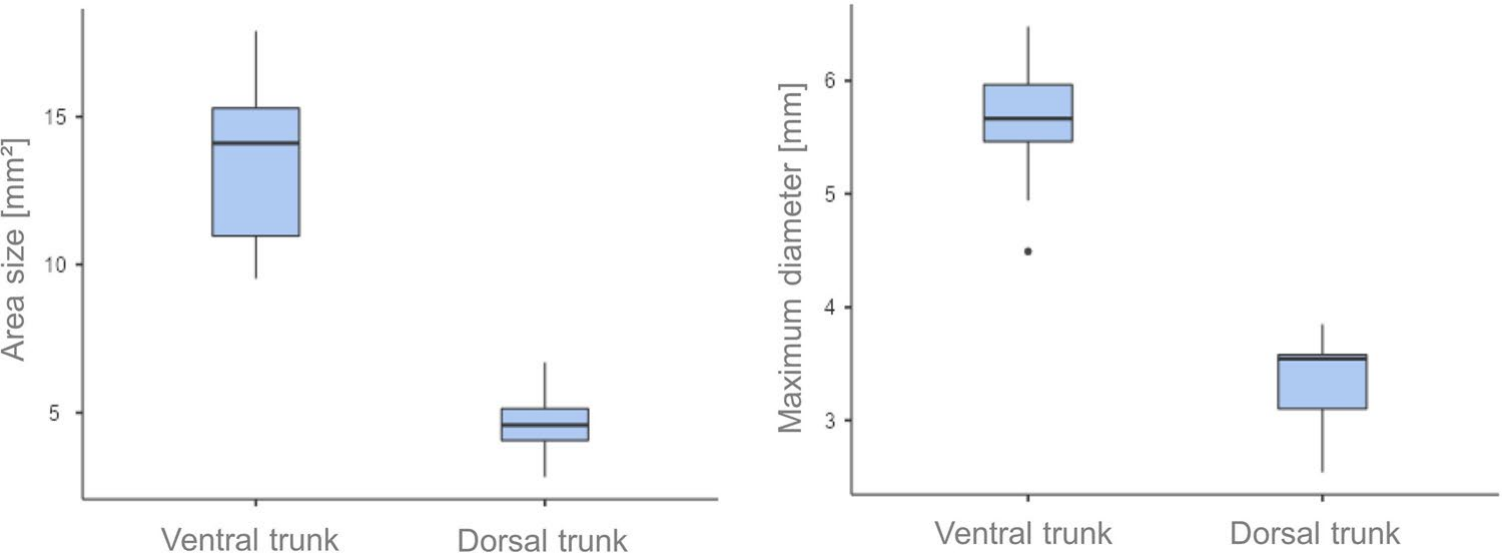
Box plots of the data from the bullet entrance holes

Exemplary photos processed by means of the image editing program are displayed in Fig. 3. The differences are obvious at first glance: both the maximum diameters and the areas of the tissue defects were larger in the specimens from the belly region compared to the back. The disparity between the two study groups is also expressed in the mean values of the size parameters as indicated in Table 1 and graphically as demonstrated in Fig. 4. Statistical evaluation confirmed that the location-dependent differences were significant on a level of less than 0.01.

**Table 1:** Maximum diameters and area sizes of the bullet entrance wounds (mean values and standard deviations)

Body region	Maximum diameter (mm)	Area size (mm^2^)
Anterior trunk	5.61 ± 0.57	13.49 ± 3.03
Dorsal trunk	3.33 ± 0.40	4.58 ± 1.17

The width and area size of the abrasion rings remained unconsidered in the given context. Nevertheless, it is worth mentioning that in general the proportion between the central skin defect and the width of the epidermis-free margin adjacent to it is shifted towards the abrasion ring in gunshot entrances with small holes as seen on the back (cf. Figure 3). A comparable relationship has been found in test shots with varying design of the bullet head [6]. In the study presented here, the epidermis (but not the underlying corium) had assumed the blue colour of haemalum, whereas the epidermis-free abrasion rings appeared pale.

**Fig. 5:**
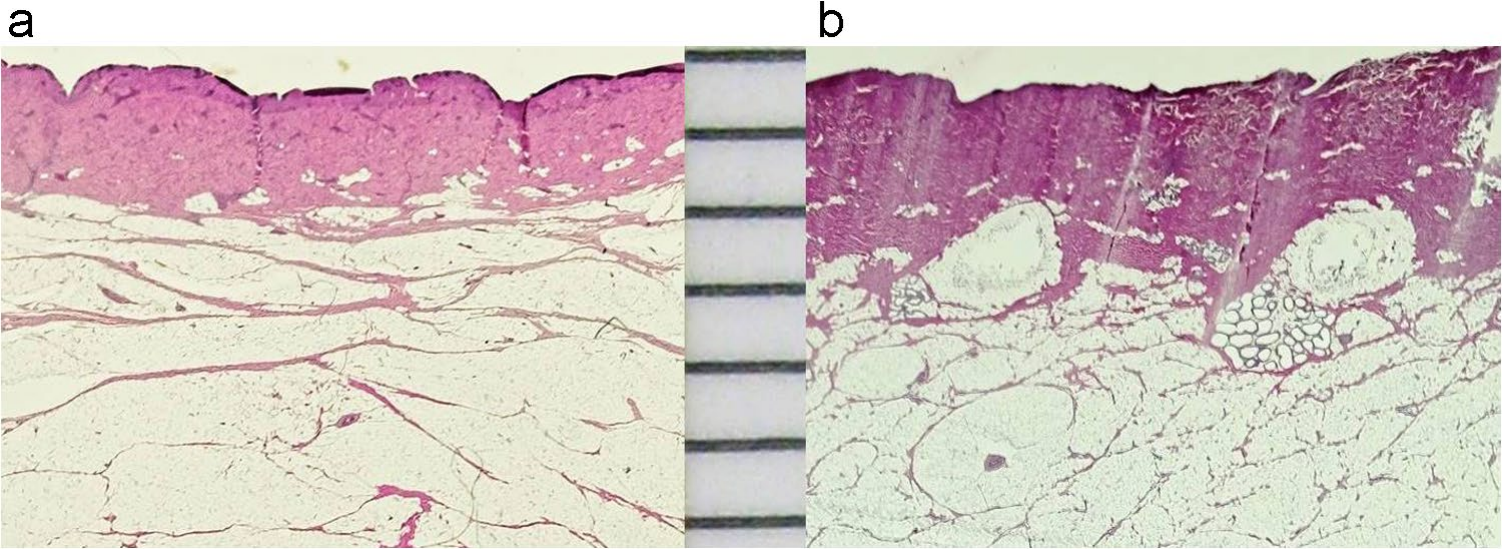
Histological sections (H&E) of porcine skin  specimens from the belly region (**a**) and the back (**b**). Note that the dermis in back skin is much thicker than in the belly region

The porcine samples subjected to histological examination revealed that the sections from the back had a distinctly thicker corium than those from the belly region (Fig. 5). The measured values were in the order of 1900 µm on the back and 1200 µm in the belly region. Taken as a whole, the results were in good agreement with the literature data for human skin samples [7, 8]. In view of the fact that the dermal thickness slightly varies within one and the same sample, it was not considered appropriate to record the data in detail.

## Discussion

The conception that a gunshot entrance hole is caused by a simple punch-out effect of the penetrating bullet has to be considered as obsolete [9]. According to the studies conducted by Sellier [10, 11] in the late 1960s, only the central parts of the impacted skin are moved into the depth of the wound channel. The adjacent tissue is radially accelerated so that the skin hole temporarily exceeds the cross-section of the entering bullet. In elastic materials such as the human skin, the lateral displacement is largely reversible and followed by secondary narrowing after the projectile has passed. As a result, the permanent hole seen at autopsy is mostly smaller than the bullet’s calibre.

The phenomenon of sub-calibre entrance holes is not confined to biological targets, as it also occurs in shots to any solid matter provided that its elasticity ensures secondary movement back to the initial position. Gunshots to rubber tyres, shoe soles or leather wear may serve as examples.

**Fig. 6:**
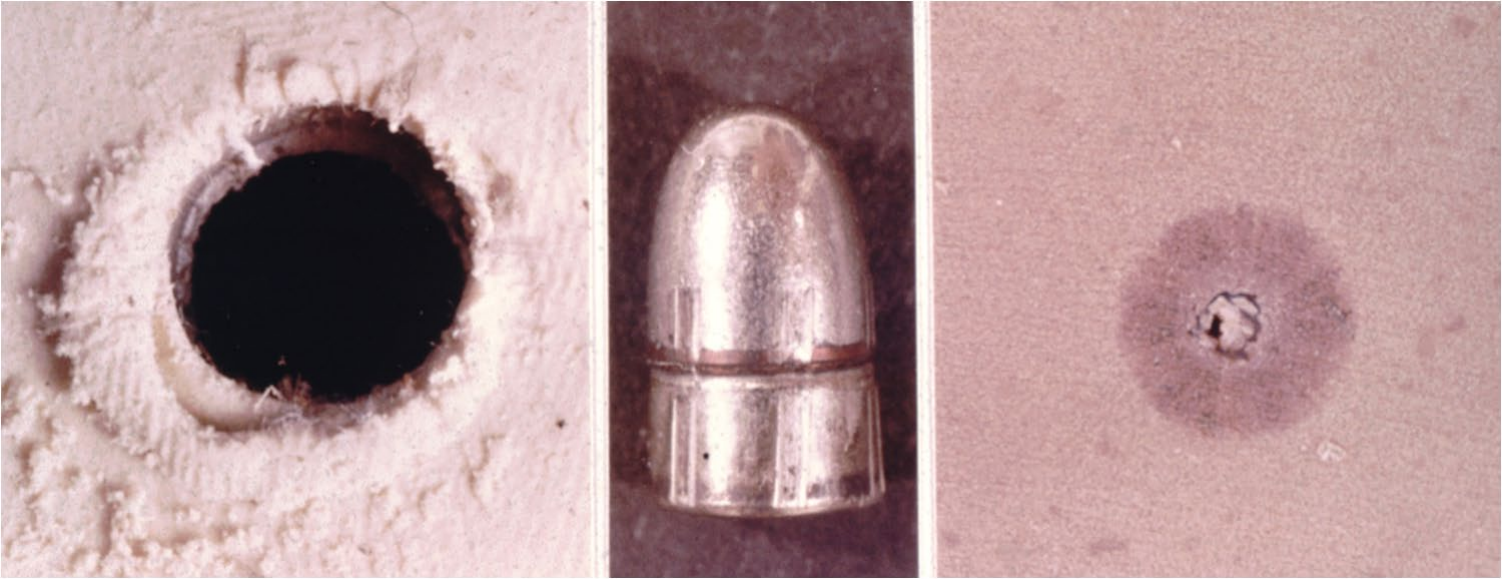
Test shots to targets having different elastic properties. On the left, the permanent entrance hole in Plasticine™ is larger than the bullet’s cross-section, whereas the size in highly elastic rubber (on the right) is much smaller than the calibre of the round-nosed projectile used (cal. .25 ACP, central picture)

In humans, the palmar and plantar regions are body areas where gunshot entrance holes are disproportionally small, obviously due to the particular stratification and elasticity of hairless skin [12]. The same applies to the toe- and fingernails consisting of keratin [13] and—inside the body—to the aortic wall [14]. On the other hand, inelastic materials such as modelling clay are characterised by a high plasticity and therefore lack a secondary narrowing of the entrance hole after the bullet has passed through (Fig. 6).

It is well known in dermatology, plastic surgery and other clinical fields that the physical properties of human skin differ subject to the body region [15]. The varying physical behaviour is to be explained by the site-specific texture, i.e. the composition of the constituent layers and their respective thickness [16]. In hairy skin having a comparatively thin epidermis, the corium seems to be of special importance, particularly since it is composed of dense connective tissue comprising collagen and elastic fibres.

In medicolegal casework, distant civilian gunshot wounds are most frequently seen in homicide victims [17–20]. Combat casualties are not taken into account in the context of this study. In perforating gunshots to the trunk, the question of the bullet’s calibre arises and—in cases with multiple hits—whether one and the same type of cartridge has been fired.

The thickness of human skin is known to vary considerably between different body regions. According to Lee and Hwang [8], the entire skin (comprising epidermis and dermis) had a mean thickness of 521 to 1977 µm in a sample of Korean subjects. The dermis alone ranged from 469 to 1942 µm on average with the highest values on the back. In comparison, the dermal thickness of the abdomen was far smaller (mean scores: 1248 µm according to [8], and 913 µm [7], respectively). Apart from the body region, some other parameters such as age, gender, posture and body mass index have to be mentioned as additional influencing factors [21, 22].

For ethical reasons, wound-ballistic examinations should not be based on experimental shots to tissues of deceased humans or live animals. During the last decades, skin from slaughtered pigs has proved as an appropriate simulant for testing the injuring effect of bullets—either in the genuine anatomical connection with deeper tissue layers (subcutis, muscles) or in composite models together with gelatine or ballistic soap [23–28]. As it turned out in several studies, human and porcine skin are quite similar regarding their anatomical structure and physical properties [29, 30]. Due to the corresponding skin stratification in both species, different features in gunshot entrance wounds such as the central tissue defect, the adjacent abrasion ring and any bullet wipe can be studied on porcine specimens.

As regards the thickness of pig dermis, Khiao In et al. [31] reported a mean value of 1620 µm for the back and 1167 µm for the caudal abdomen. According to Turner et al. [32], the dermis in the caudal back was the thickest averaging 1687 µm vs. ~ 1000 µm in the belly region. A similar proportion between the dermal thickness of back and abdomen was determined in our test material. When comparing such measurements, it has to be considered that apart from the age also the pig breed and the kind of farming may affect the results [33, 34].

To the best of our knowledge, the correlation between the body region hit by a bullet and the size of the resulting entrance hole in skin has never been the subject of systematic test shots in an experimental setting. In 2005, Bir et al. [35] have shown that the energy density required for 50% risk of skin penetration by missiles was significantly lower on the anterior thorax in comparison to the posterior thorax.

The investigation presented here compared distant shots to the belly region and back, as these parts of the body are known to have different thicknesses of the dermis so that effects on the physical properties of the skin are to be expected. As already mentioned above, the permanent size of the bullet entrance hole is mainly determined by the elastic resetting forces which secondarily narrow the gap left by the passing bullet.

In the present study, the test shots were fired from a semi-automatic pistol using cartridges 9 × 19 mm Luger, a widespread handgun ammunition employed both for civilian and military purposes. In our test series, full metal jacket projectiles with a round nose design were shot at the skin specimens. In previous investigations, it had been shown that the entrance holes caused by round-nosed bullets are smaller than those from projectiles with a truncated cone or a wadcutter configuration [6].

Distant shots to porcine skin revealed that under otherwise identical conditions the bullet entrance holes differed significantly in size: on the back, both the diameters and the areas of the skin defects were considerably smaller in comparison to the belly region. An accordant disparity can be encountered in forensic practice as exemplarily shown in Fig. 1. Due to the great number of variables (as to the victim’s age, any intermediate target such as clothing, the specific body region affected, the angle of shot and many others), real autopsy cases do not represent a sufficiently homogenous material for statistic evaluations, even if the examined injuries were produced by one and the same kind of weapon and ammunition. In contrast, porcine skin can be collected from well-defined body regions of slaughtered pigs having a given age and nutritional status. All other parameters (firing distance, orthogonal direction of shot, identical lot of ammunition etc.) can be standardised as well. In view of the marked differences between the body regions examined, the limitation to a single, randomly selected test animal seems acceptable.

The differing skin structure on the dorsal and ventral trunk is the most plausible reason for the disparity between the sizes of bullet entrance holes. As outlined above, the various thickness of the dermis comprising connective tissue fibres largely determines the skin’s physical properties and especially its elastic behaviour. The smaller size of entrance holes on the dorsal trunk can be explained by the fact that the comparatively thick dermis of the back undergoes a mainly elastic deformation when passed by the bullet. As a consequence, the skin hole secondarily narrows due to the elastic recoil of its margins.

The results obtained reinforce the view that it is not possible to draw conclusions about a bullet’s calibre from the size of a gunshot entrance hole in skin. Furthermore, it should be noted that differences in size measured in various body regions do not necessarily imply that the wounds were caused by projectiles of unequal calibres.

## Conclusions

- In medicolegal casework, bullet entrance holes located in different body regions often vary in size, even if they were caused by the same weapon and ammunition.

- Distant shots fired to porcine skin serving as a surrogate for human skin showed that the entrance holes on the back are significantly smaller than those in the belly region.
- The location-dependent size of bullet holes can be explained by the various thickness of the dermis which decisively determines the physical properties of the skin and its elastic deformation at the penetration site.
